# Cross-reactive LTP sensitization in food-dependent exercise-induced urticaria/anaphylaxis: a pilot study of a component-resolved and in vitro depletion approach

**DOI:** 10.1186/s13601-016-0136-5

**Published:** 2016-12-22

**Authors:** Diana Margarida Gonçalves Solha Pereira da Silva, Teresa Maria Silva Vieira, Ana Maria Alves Pereira, André Miguel Afonso de Sousa Moreira, José Luís Dias Delgado

**Affiliations:** 1 Serviço de Imunoalergologia, Centro Hospitalar São João, Porto, Portugal; 2 Laboratory of Immunology, Basic and Clinical Immunology Unit, Faculty of Medicine, Porto University, Porto, Portugal; 3 Unidade de Imunoalergologia, Unidade Local de Saúde do Alto Minho, Viana Do Castelo, Portugal; 4 Department of Clinical Pathology, Centro Hospitalar São João, Porto, Portugal; 5 ISPUP-EPIUnit, Universidade do Porto, Porto, Portugal; 6 CINTESIS and Biostatistics and Medical Informatics, Faculty of Medicine, University of Porto, Porto, Portugal

**Keywords:** Immunodepletion, Food-dependent exercise-induced anaphylaxis, Urticaria, Lipid transfer proteins, Depletion, In-vitro diagnosis

## Abstract

**Background:**

Challenge tests for food-dependent exercise-induced anaphylaxis (FDEIA) carry some risk and have a high rate of false negatives. Our aim was to explore the usefulness of an in vitro immunodepletion assay and an allergen microarray test in the identification of IgE-mediated cross-reactive food allergens in patients with suspected FDEIA or food-dependent exercise-induced urticaria and panallergen sensitization.

**Methods:**

Three patients with a history of food dependent exercise induced urticaria/anaphylaxis and food panallergen sensitization in whom a food-exercise challenge was not feasible were selected: a 25-year-old man with cholinergic urticaria who experienced generalized urticaria and angioedema during a soccer match after drinking a peach-based soft drink; a 19-year-old woman with allergic rhinitis and controlled asthma who experienced anaphylactic shock while playing soccer, having eaten walnuts in the previous 90 min; and a 57-year-old man with baker’s asthma who experienced four episodes of anaphylaxis during exercise after ingesting wheat-containing food. All individuals underwent a diagnostic work-up with skin prick tests, specific IgE (sIgE) and ImmunoCAP ISAC test. For the in vitro immunodepletion procedure, patients’ serum was pre-incubated with the suspected native allergen (peach, walnut, or wheat) in solid phase (ImmunoCAP). The eluted serum, containing unbound IgE, was collected and samples were re-tested using Immunocap ISAC 112 and compared with baseline results.

**Results:**

All individuals were sensitized to lipid transfer proteins. The first patient was sensitized to Pru p 3, Cor a 8, Jug r 3, and Ara h 9; after pre-incubation with peach there was 100% depletion of sIgE to all components. The second patient was sensitized to Pru p 3, Cor a 8, Jug r 3, and Ara h 9; immunodepletion with walnut depleted sIgE to Ara h 9 by 67%, Pru p 3 and Pla a 3 (60%), Art v 3 (75%), Jug r 3 (88%), and Cor a 8 (100%). The third patient was sensitized to Pru p 3, Jug r 3, Ara h 9, and Tri a 14; immunodepletion with wheat depleted Tri a 14 only (100%).

**Conclusions:**

In vitro immunodepletion might be a useful diagnostic tool in food dependent exercise induced urticaria/anaphylaxis with panallergen sensitization, particularly for identifying the culprit allergen and guiding dietary elimination recommendations.

## Background

Exercise induced anaphylaxis is a potentially fatal clinical syndrome in which anaphylaxis is triggered by mild to vigorous exercise [1, 2]. The pathophysiological mechanisms underlying this disease have not been fully demonstrated [3]. When food is involved as a co-factor, the condition is called food-dependent exercise-induced anaphylaxis (FDEIA), and it can be further classified according to the trigger food(s) [1, 4]. Episodes occurring after the ingestion of certain foods are described as specific FDEIA, while those occurring after the ingestion of any food are described as non-specific FDEIA [5]. Frequently, mild physical activity can trigger severe systemic reactions and some patients experience mild-moderate systemic allergic reactions with exercise, dependent on food ingestion [6]. These milder reactions have been recently reported as food dependent exercise-induced urticaria/angioedema, both associated with lipid transfer protein [7] and with wheat [8]. Several foods are involved, wheat is the most commonly reported, namely in Japan [9], but also seafood, vegetables, fruits and nuts [5, 9]. Geographical differences occur in the implicated food, shellfish or soy were more frequently reported in Asia [9–11], fruits and vegetables in the Mediterranean area [12, 13]. Multiple food hypersensitivity is reported in a large percentage of individuals with FDEIA, who also have a high rate of sensitization to panallergens, such as lipid transfer proteins (LTPs) [12].

Diagnosis is highly dependent on a thorough clinical history including a detailed description of all food ingested before and after the physical activity that triggered the anaphylactic reaction [5, 9]. Romano et al. [12, 14], suggested to use a combination of in vivo tests, (skin prick tests [SPTs] and prick to prick tests [SPPT] to a wide panel of allergens, chosen accordingly to the clinical history) and in vitro tests, including recombinant allergens. Challenge tests are needed to provide a definite diagnosis and should include a food challenge, an exercise challenge, and a combined food-exercise challenge [9]. False-negative results can occur, however, as food-exercise challenges fail to confirm diagnosis in up to 30% of patients [9, 14]. False negatives can be explained by the unpredictability of FDEIA, as it can occur during exercise of different intensities and at varying periods of time after food intake; other contributing cofactors [15] include stress, drugs (e.g., anti-inflammatories), menstruation, and weather [13, 16], namely seasonal pollen exposure in pollen sensitized individuals with cross-reactivity with food allergens [13] and environmental temperature variations [16, 17]. Diagnosis is even more complex in patients with multiple food hypersensitivity.

Numerous food-exercise challenges may be needed to identify the cause of FDEIA, particularly in cases of multiple food sensitization. This approach is obviously time-consuming, carries the risk of multiple reactions, and is not always feasible [12–14]. While component-resolved diagnosis can be used to identify primary and cross-reactive allergenic compounds involved in polysensitization and guide which foods should be avoided in a challenge [12], it does not resolve the problems related to exercise challenges. Until now, the most adequate method for FDEIA suspicion diagnosis is a complete anamnesis followed by an exercise challenge that gathers, as far as possible, all the characteristics and co factors that elicited the reaction [18]. New, safer, and more specific diagnostic tools are needed to identify the FDEIA triggers and establish preventive measures. The aim of this study was to explore the usefulness of an in vitro immunodepletion assay and an allergen microarray to identify IgE-mediated cross-reactivity between food allergens in three patients with suspected food-dependent exercise-induced urticaria/anaphylaxis and pan-allergen sensitization.

## Methods

### Patient selection and study design

We performed a pilot study of three patients with a clinical history of food-dependent exercise-induced urticaria/anaphylaxis and sensitization to food panallergens in whom a complete diagnostic work-up including a food-exercise challenge was ruled out for clinical reasons. Immunodepletion was performed using the serum of each patient, and we compared results for native and depleted serum to assess cross-reactivities with the main suspected trigger food.

Patient I is a 25-year-old man with a previous history of cholinergic urticaria and mild oral allergy syndrome to peach developed generalized urticaria, lip swelling, and facial angioedema 30 min after a recreational soccer match. He required medical attention and was treated with systemic steroids and antihistamines within 45 min. Adrenaline was not administered and the patient fully recovered within 2 h. He recalled drinking a peach-based soft drink just before the match. The patient, denied previous episodes of facial angioedema or lip swelling. A food-exercise challenge was ruled out due to the difficulty of interpreting signs and symptoms during the challenge (the patient frequently experienced cholinergic urticaria during exercise).

A 19-year-old woman, patient II, with controlled asthma and a previous history of allergic rhinitis to mites and grass pollens experienced anaphylactic shock during a soccer match. She had eaten walnuts 90 min before the match and tomato, mango, orange, wheat bread with cheese in the preceding 6 h. She developed urticaria, generalized pruritus, facial edema, and dyspnea, followed by a loss of consciousness. On admission to the emergency department at the local hospital, she was hypotensive (70/40 mmHg), hypoxic (peripheral saturation of 84%), and had peripheral cyanosis. Treatment with epinephrine, corticosteroids, bronchodilation, and fluid therapy led to full recovery within 24 h. A food-exercise challenge was not performed because of the severity of her reaction.

A 55-year-old man, patient III, with a past history of ischemic heart disease, hypertension, type 2 diabetes, and a previous history of baker’s asthma and occupational rhinitis on exposure to cereal flour reported four episodes of anaphylaxis in the previous 6 months. His daily medications included amlodipine 5 mg, indapamide 1.5 mg, acetylsalicylic acid 100 mg, simvastatin 40 mg, gliclazide 20 mg, metformin 850 mg, budesonide 400 µg via a dry powder inhaler, and montelukast 10 mg. The anaphylactic reactions had occurred after hiking or brisk walking. The patient had eaten grapes and bread before the second episode and pasta and meat before the last one. He could not recall what he had eaten in the other two episodes.

### Skin testing

Skin testing was performed according to the European Academy of Allergy and Clinical Immunology guidelines [9]. All patients underwent SPT with commercial extracts of the following aeroallergens: *Dermatophagoides pteronyssinus*, *Dermatophagoides farinae*, *Lepidoglyphus destructor*, *Felis domesticus*, *Canis familiaris*, *Platanus acerifolia*, *Betula verrucosa*, *Olea europaea*, grass mixture (*Dactylis glomerata*, *Festuca elatior*, *Lolium perenne*, *Phleum pratense* and *Poa pratensis*), weed mixture (*Artemisia vulgaris*, *Chenopodium album*, *Parietaria judaica*, and *Plantago lanceolata*), *Cladosporium herbarum*, and *Aspergillus fumigatus*.

Food allergy tests were performed according to each patient’s clinical history with commercial food extracts with SPTs and fresh foods with SPPT. The results are summarized in Table 1. SPTs with purified natural date palm profilin (ALK-Abelló, Denmark) and peach, containing only LTP (Pru p 3, 30 mg/mL; ALK-Abelló, Denmark) were performed as appropriate.

**Table 1:** Results of skin tests and specific IgE work-up study for each patient accordingly to their clinical history

Patient	SPT	SPPT	sIgE (kU_A_/L)
I Total IgE = 14kU/L	LTP—pos. Profilin—pos.	n.a.	Pru p 1 < 0.35 Pru p 3 = 11.00 Pru p 4 < 0.35 Apple = 4.21
II Total IgE = 297kU/L	Apple—neg. Peach— neg. Orange— pos. Strawberry—pos. Banana— neg. Kiwi—neg. Tomato—neg. Hazelnut—pos. Peanut—neg. Almond—neg. Soy— neg. Oat—neg. Maize—neg. Rye—neg. Wheat—neg. Cow milk—neg. Egg yolk—neg. Egg white—neg.	Apple—pos. Peach—neg. Orange—pos. Strawberry—neg. Banana—neg. Kiwi—pos. Mango—neg. Tomato—neg. Hazelnut—pos. Walnut—pos. Peanut—neg.	Apple = 7.97 Orange = 3.83 Walnuts = 21.10 Strawberry = 13.10 Banana = 4.26 Kiwi = 3.36 Tomato = 1.28 Mango = 0.69 Maize = 11.30 Peanut = 7.07
III Total IgE = 280kU/L	Wheat—pos. Peanut—neg. Soy—neg. Cow’s milk—neg. Egg white—neg. Cod—neg.	Wheat—pos	Wheat = 0.96 α—amylase < 0.10 Gliadin < 0.10 ω-5-gliadin (Tri a 19) < 0.10 egg white < 0.10

*LTP* lipid transfer protein, *n.a.* not available, *neg.* negative, *pos.* positive, *sIgE* specific IgE, *SPT* skin prick test, *SPPT* skin prick to prick test

Histamine hydrochloride 10 mg/mL and sodium chloride 0.9% were used as the positive and negative controls, respectively. Disposable 1 mm tip lancets were used. A positive skin prick test was defined as a largest wheal diameter of ≥3 mm.

### Specific IgE and microarray-based IgE detection

Blood samples were collected after the first visit and stored at −20 °C until assayed. Total serum IgE and specific IgE (sIgE) to allergen extracts were measured using ImmunoCAP (Thermo Fisher Scientific, Uppsala, Sweden). sIgE values greater than 0.35 kU/L were considered clinically relevant and positive.

In all patients a multiple allergen component analysis was performed with native and allergen-depleted serum using the ImmunoCAP ISAC 112 microarray test (Thermo Fisher Scientific, Uppsala, Sweden). The results were analyzed on a semiquantitative basis and expressed as ISAC Standardized Units (ISU-E) (range 0.3–100 ISU-E).

### Immunodepletion procedure

Immunodepletion was performed, as previously described [19, 20]. For depletion procedure, IgE antibodies bound to an immobilized allergen or allergen extract, in this study to an ImmunoCAP matrix, are removed from the serum after incubation step and then analyzed. Using the serum of each patient and a solid-phase Immunocap for the main allergen suspected in each patient: peach (f 95) in patient I; walnut (f 256) in patient II and wheat (f 4) in patient III. The procedure was performed in duplicate, using two controls. Each ImmunoCAP, was pre-washed four times: twice with Immunocap washing solution two times and twice with phosphate buffer at neutral pH. Then, 50 µL of serum sample was added to each pre-washed ImmunoCAP and incubated for 60 min at room temperature. The ImmunoCAP was then centrifuged at 1450*g* for 2 min and the depleted serum of each patient (containing unbound IgE) was collected, pooled, and frozen at −20 °C. The depleted and native sera were then analyzed in parallel using the ImmunoCAP ISAC microarray, as previously described. The depletion percentage was calculated as the ratio between the results (ISU-E) for each allergen component for the depleted and native serum samples.

## Results

Patient II had positive SPTs to house dust mites (*D. pteronyssinus, D. farinae*, *and L. destructor*) and the grass pollen mix. These results were consistent with the ImmunoCAP ISAC results, which showed the following sensitizations (in ISU-E): Der p 1, 27.0; Der p 2, 28.0; Der f 1, 12.0; Fel d 1, 5.0; Can f 1, 1.9; Phl p 4, 0.7; Phl p 1, 0.4; and Cyn d 1, 0.5. None of the other patients showed sensitization to any other aeroallergens or food allergens than those specified in Tables 1 and 2 by SPTs, sIgE and in the ISAC profile.

**Table 2:** Results of ImmunoCAP ISAC microarray test with native and depleted serum (before and after immunodepletion); pre-incubation performed with peach (f 95) in patient I, with walnut (f 256) in patient II, and with wheat in patient III (f 4)

	Patient I			Patient II			Patient III		
	Native serum	Depleted serum	Depletion (%)	Native serum	Depleted serum	Depletion (%)	Native serum	Depleted serum	Depletion (%)
*LTP*									
rPru p 3	10.0	0.0	100	14.0	4.6	67	0.7	0.8	0
rPla a 3	8.6	0.0	100	7.8	2.6	67	*nd*	*nd*	–
rCor a 8	7.7	0.0	100	0.7	0.0	100	*nd*	*nd*	–
rAra h 9	6.8	0.0	100	6.2	2.5	60	2.5	2.6	0
nJug r 3	5.7	0.0	100	15.0	1.8	88	1.4	1.5	0
nArt v 3	1.4	0.0	100	1.6	0.4	75	0.9	0.9	0
rTri a 14	*nd*	*nd*	–	*nd*	*nd*	–	0.4	0.0	100
*PR*-*10*									
rMald 1	*nd*	*nd*	–	*nd*	*nd*	–	0.4	0.4	0
rPru p 1	*nd*	*nd*	–	*nd*	*nd*	–	0.4	0.4	0

Data are presented in ISAC Standardized Units unless otherwise specified

*nd* not detected

Patient I had positive sIgE to the peach component Pru p 3 and apple (Table 1). ImmunoCAP ISAC 112 showed sensitization to LTP components from food-derived allergens (Pru p 3, Cor a 8, Ara h 9, and Jug r 3) and aeroallergens (Pla a 3 and Art v 3) (Table 2). Patient II was sensitized to multiple fruits and nuts, and the STP and prick to prick test results were consistent for orange and hazelnuts. The SPTs, SPPTs and sIgE determination showed sensitization to strawberry, apple, kiwi fruit, tomato, mango, maize, walnut and peanut. Sensitization to the food allergens was suspected to be mediated by LTP in all cases, with the strongest sensitization observed for the main suspect, walnut (Jug r 3), followed by peach (Pru p 3), peanut (Ara h 9), and hazelnut (Cor a 8). As expected given his previous history of baker’s asthma, patient III showed sensitization to wheat in both the SPT and sIgE determination. The ImmunoCAP ISAC 112 results showed that sensitization to wheat was mediated by LTP (Tri a 14) but not by ω-5-gliadin. Additional sensitization to other LTP allergens was also found, namely to Pru p 3, Ara h 9, and Jug r 3.

**Fig. 1:**
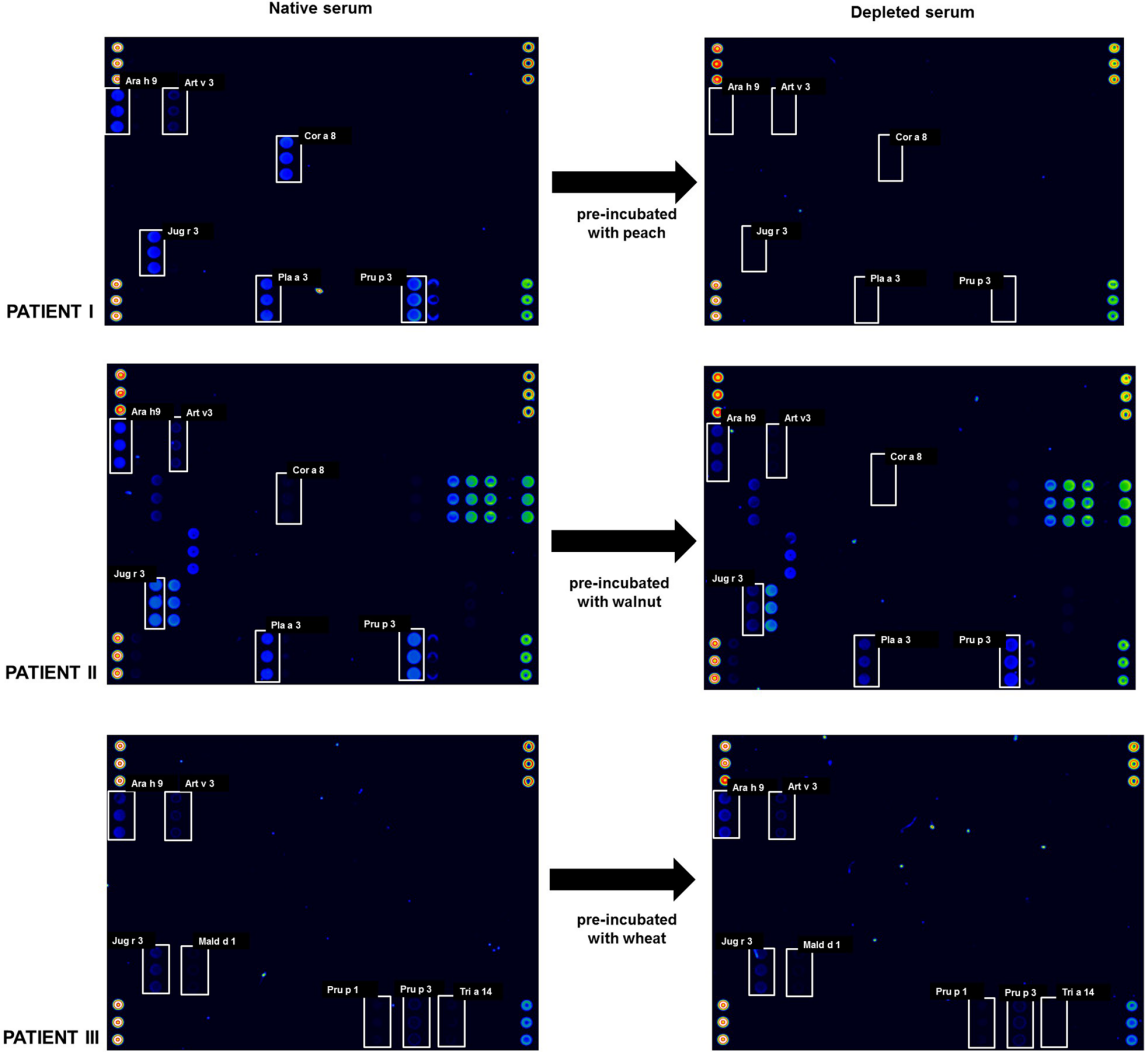
ISAC microarray results comparing native and depleted serum for each patient. The suspected culprit allergens in each case are shown in *white boxes*. Serum was pre-incubated with ImmunoCAP peach (f 95) in patient I, walnut (f 256) in patient II, and wheat (f 4) in patient III

Evaluation of cross-reactivity by immunodepletion (Fig. 1) showed varying results. In patient I, whose serum has been pre-incubated with the solid-phase ImmunoCAP ISAC peach extract, 100% depletion was seen for all LTP related foods and aeroallergens. Some reactivity was also seen for the 2S albumin Ses I 1 (1.9 ISU-E), that remained positive (1.4 ISU-E) after the immnodepletion (Fig. 1). High depletion percentages, in the range of 60-100%, was also observed for patient II, whose native serum had been pre-incubated with walnut. Depletion levels were highest for Cor a 8 (100%), possibly due to the lower sIgE levels in the native sample; Jug r 3 depletion was 88%. Finally, in the case of patient III, whose serum had been pre-incubated with wheat, we observed 100% depletion for Tri a 14. No other changes were observed.

As the gold standard diagnostic test for FDEIA—a food-exercise challenge—was not an option in any of these cases, the patients were advised to eliminate certain foods from their diet based on their clinical history, the in vitro results, and guideline recommendations [11]. Therefore, patient I was advised to avoid peach, the suspected culprit allergen, as well as hazelnut, peanut, and walnut before exercise. Due to the severity of her anaphylactic reaction and the high depletion percentages observed, patient II was advised to avoid walnut, hazelnut, peanut, and peach. Finally, patient III was advised to avoid foods containing wheat before exercise. All patients, except patient II, which decreased exercise practice by avoiding soccer matches due to fear of another reaction, resumed their regular physical activity following the stated recommendations. After a three year follow-up, none of the patients have experienced any episodes of anaphylaxis since these recommendations were implemented.

## Discussion

We have presented three clinical reports of suspected food-dependent exercise-induced urticaria/anaphylaxis in which LTP sensitization was a common feature. We have also shown, for the first time, that individual cross-reactivity patterns can be evaluated using in vitro immunodepletion with the suspected solid-phase allergen extract.

While a food-exercise challenge is the gold standard for diagnosing FDEIA, it only confirms diagnosis in up to 70% of patients, including those with reproducible and recurrent clinical FDEIA [9]. This lack of sensitivity is related to the difficulty of replicating the conditions in which the reaction occurred, such as the exercise environment [16, 17], the ovulatory phase [21] and concomitant use of drugs, such as aspirin [22]. In a recent study designed to improve diagnostic accuracy, exercise challenges were performed after the patients had ingested the suspected foods along with aspirin [23]. While the approach did prove to be more accurate, it was associated with more severe reactions, with 20% of patients requiring adrenaline [23]. Food-exercise challenges are particularly challenging when several foods are suspected, or in patients with sensitization to panallergens, and/or co-morbidities, such as ours. The findings of this pilot study suggest that in vitro assays might be useful for component-resolved diagnostic testing of major sensitizers.

Sensitization to LTP from both fruits [24, 25] and tree nuts [25] is high in the Mediterranean area, as was recently shown for hazelnut in the EuroPrevall study [26]. LTP sensitization is frequently associated with severe systemic reactions [25]. In a recent study of a large series of patients with FDEIA from the Mediterranean area, LTPs were found to be the most frequent sensitizers [12], supporting previous reports of patients showing sensitization to LTPs from several different foods [27, 28]. Multiple food hypersensitivity is a hallmark of FDEIA [12] and poses major diagnostic challenges.

In-vitro diagnostic testing, with component-resolved diagnosis or the ImmunoCAP ISAC allergen microarray has proven useful for assessing individual risk of anaphylaxis [29] and investigating idiopathic anaphylaxis [30]. Although the use of recombinant food allergen proteins can help to understand cross-reactivity between unrelated plant species, clinical symptoms are frequently heterogeneous [31] and clinically irrelevant sensitization also occurs, particularly in LTP-sensitized patients [32]. In a study of patients with sIgE to LTP-containing foods (e.g., apple, hazelnut, walnut, peanut, and tomato), a variety of clinical symptoms, ranging from none to systemic, was reported [32]. In a recent study by Pascal et al. it was shown that asymptomatic sensitization was common and that the use of specific IgE testing by microarray failed to discriminate allergic versus tolerant individuals [33]. A broad sensitization LTP-sensitization profile was also observed in the three patients described in this paper, even though the clinical signs and symptoms pointed to a specific culprit food. Without further investigation, such patients could be subjected to unnecessary dietary elimination or to multiple food-exercise challenges, which are time-consuming, associated with a considerable rate of false negatives, and of course not without risk. In such cases, the use of other specific diagnostic tests, such as serum inhibition assays, the basophil activation test, and the histamine release assay [34] may be useful to guide clinical recommendations.

We used an immunodepletion procedure with solid-phase allergen extracts (ImmunoCAP) to investigate individual cross-reactivity profiles. Previous studies have used inhibition procedures for this purpose [27, 28, 35, 36]. The authors of one study of apple-allergic patients with oral allergy syndrome or systemic symptoms found several patterns of sensitization, and reported LTP to be the most prevalent sensitizer in patients with systemic symptoms. In individuals sensitized to LTP only, the inhibition assay indicated high cross-reactivity between Mal d 3 and Pru p 3. Similar results were found for individuals sensitized to both LTP and profilins, although the inhibition rates were lower [27]. We observed high depletion rates for all foods and aeroallergens tested against peach in patient I. This was not the case, however, for patient II, who experienced a severe anaphylactic reaction, despite co-sensitization to aeroallergen and PR-10 components.

High cross-reactivity with the peach LTP, Pru p 3, was observed in all three patients studied, which is consistent with results for previous studies of tomato [28], mulberry [35], hazelnut and cherry [36]. Cross-reactivity patterns, however, can vary according to the food involved. In a study of celery stalk sensitization mediated by LTP (Api g 2), patients who had clinical symptoms on eating celery had higher self-inhibition to Api g 2 than those who were sensitized but had no symptoms; the asymptomatic group, by contrast, had stronger Pru p 3 and Art v 3 reactivity [37]. LTPs from different foods react differently. The strawberry LTP, rFra a 3, for instance, has been shown to have less allergenic potency than peach or apple and does not appear to be associated with clinical relevance [38]. Therefore, strawberries might be tolerated by Pru p 3-sensitized individuals with clinical symptoms. In a series of LTP-monosensitized allergic patients, food-specific IgE levels showed a hierarchical order, with peach in the first place, followed by apple, walnut, hazelnut, peanut, lentil, maize, soybean, tomato, kiwi, sesame, mustard, melon, and celery [39]. Sensitization, however, did not necessarily result in clinical symptoms, as was the case with the majority of patients sensitized to lentil, maize, or soybean. Inhibition studies assessing cross-reactivity profiles (both intensity and patterns) as well as immunodepletion assays might thus be helpful for supporting a clinical history, as sensitization does not always equate to clinical allergy.

We advised our three patients to avoid nuts, including peanuts, even though they had never experienced an allergic reaction to these foods. One study of LTP-monosensitized patients with allergic reactions to peach showed that half of the patients with co-sensitization to peanut were clinically allergic to it [40]. In another study with a similar population, those with clinical reactions to peanut had higher levels of sIgE than those without, but there was no difference in the prevalence of local versus systemic reactions [41]. Although it is highly likely that Ara h 9 is present in peanut extract [42], neither of the studies reported sensitization to this LTP or correlated it with clinical symptoms. In the presence of a history of a severe allergic reaction, sIgE levels to peanut would appear to only partially predict clinical relevance [41]. In a study of component-resolved IgE profiles, 10% of peanut-allergic patients showed sensitization to Ara h 9 [43]. A strong correlation has also been found between Ara h 9 and Pru p 3 sensitization, although Pru p 3 probably acted as the primary sensitizer [43]. This cross-reactive sensitization had clinical relevance, justifying the need for the elimination of these foods from the diets of those affected. This might not, however, be the case with other food allergens.

Wheat LTP have been identified as a major allergen associated with baker’s asthma [44] and has also been linked to anaphylaxis induced by flour-derived foods [45]. In one inhibition study, cross-reactivity between peach Pru p 3 and the wheat LTP Tri a 14 was very limited in individuals with baker’s asthma [44]. In another series of eight patients who experienced anaphylaxis after eating wheat flour–derived foods, six were also sensitized to Pru p 3 or Art v 3 and reacted to other plant foods, although only two were specifically sensitized to recombinant Tri a 14 [45]. No inhibition or depletion procedures were performed in this group of patients. In our series, although patient III was sensitized to other LTP food components, depletion was seen only for wheat, which might be explained by the mild sensitization to Tri a 14, which would have been easily depleted, and by the fact that the wheat sIgE extract may have had a low quantity of LTP components, thereby insufficient to deplete the other LTP components. In a study of three patients with wheat-dependent exercise-induced anaphylaxis due to Tri a 14 and a history of severe peach allergy, cross-reactivity between peach and wheat LTP was relatively weak, and the authors inferred that only a small percentage of patients allergic to peach LTP have wheat allergy [46]. Basophil activation tests have proven useful for the in vitro diagnosis of wheat-dependent exercise-induced anaphylaxis [47], in terms of identifying both patients and the causative allergen (hydrolyzed wheat protein) [48]. However, the sensitivity and specificity of these tests have not been established [34].

Our study has some limitations. The use of different allergen extracts (ImmunoCAP) for each patient is one limitation, for example, because like with inhibition assays, the potency for depletion may vary between extracts. Higher depletion rates were observed for patient I probably because the peach extract coupled to the Immunocap has a higher LTP content (Pru p 3) or because this extract might cause greater inhibition of other LTP-related components than walnut or wheat extracts, which were associated with lower depletion rates. One way to overcome this limitation would be to perform the assay using all the peach, wheat, and walnut extracts studied in each patient. Nevertheless, such an approach would have diverted the investigation from the individual culprit allergens, as patients I and II, for example, were not found to be sensitized to wheat. A second limitation of our study is the possibility that untested/not serologically identified allergens might have been involved in the reactions, as we observed inconsistent results for SPTs, prick to prick tests, and specific IgE determination in all three patients. This is, however, unlikely, as none of the patients have experienced any allergic reactions since we recommended the dietary eliminations. Finally, because our study was based on real-life cases, we did not follow the same diagnostic protocol for all patients. The diagnostic work-up was adapted to each patient’s complaints and performed at different points in time. However, this is unlikely to have affected our main results, as the multiplex studies were performed in parallel at the same time. Our results cannot be generalized due to the small number of cases studied and the lack of knowledge on the accuracy of the diagnostic tests. Notwithstanding, we believe that our approach might be helpful in similar cases when food-exercise challenges are contraindicated or unfeasible, e.g., in patients with panallergen sensitization or sensitization to several suspected foods. Our results focused in LTP sensitization, but this evaluation could also be used for patients sensitized PR-10, namely those with severe allergic reactions with soy consumption which have cross-reactivity to Bet-v1 homologues [49], or patients sensitized to storage proteins in order to evaluate cross reactivity between nuts [50], particularly when anaphylaxis is dependent on other co-factors [51]. This was a pilot study and further application of the diagnostic tests described requires comparison with the gold standard food-exercise challenge for each food in order to validate this approach. Nevertheless, the ethical implications of such a study should be carefully discussed, as several food-exercise challenges would be necessary, and these have a diagnostic accuracy of well below 100% and are not free of risk.

## Conclusions

The diagnosis of food-dependent exercise-induced urticaria/anaphylaxis is challenging and the gold standard test, food intake followed by an exercise challenge, has a high rate of false negatives and entails risk for the patient. We have presented a translational pilot study in which we used an in vitro immunodepletion procedure to guide individual dietary elimination recommendations. None of the patients have experienced any anaphylactic or allergic reaction episodes with exercise since these recommendations were made. The immunodepletion assay also proved to be suitable as a diagnostic tool and helped to understand cross-reactivity patterns in individuals with food-dependent exercise-induced urticaria/anaphylaxis who were sensitized to the LTP panallergen. Although application in clinical practice is limited by the small number of cases studied and the need for validation, the technique appears to be a promising, simple, and easy tool, which associated with a thorough clinical history, might guide diagnosis and treatment recommendations.

## Abbreviations

FDEIA: food-dependent exercise-induced anaphylaxis
LTP: lipid transfer protein
sIgE: specific IgE
SPTs: skin prick tests
SPPTs: skin prick to prick tests
